# Case Report: Bradycardia in neonatal lupus: differential diagnosis between atrioventricular block and premature atrial contractions with block

**DOI:** 10.3389/fped.2024.1337135

**Published:** 2024-07-31

**Authors:** Wei-Li Liu, Ying-Hsuan Peng

**Affiliations:** ^1^ Department of Pediatrics, Dalin Tzu Chi Hospital, Dalin, Chiayi County, Taiwan; ^2^ Department of Pediatrics, Chung Shan Medical University Hospital, Taichung, Taiwan; ^3^ Department of Pediatrics, School of Medicine, Chung Shan Medical University, Taichung, Taiwan

**Keywords:** neonatal lupus, bradycardia, high-degree atrioventricular block, premature atrial contraction with block, atrial tachycardia

## Abstract

Neonatal lupus may be associated with severe cardiac conduction problems, including high-degree or complete atrioventricular (AV) block, necessitating immediate pacemaker implantation during the neonatal period. However, cardiac manifestations of neonatal lupus may extend beyond AV block. Our case was a full-term female neonate, who presented with fetal arrhythmia and bradycardia with a heart rate of approximately 70–75 beats per minute after birth. Neonatal lupus was diagnosed later due to positive maternal and neonatal anti-SSA/Ro antibody. High-degree AV block was considered initially but bigeminy premature atrial contractions (PACs) with block was confirmed through a detailed evaluation of an electrocardiogram, which demonstrated unfixed PP intervals and fixed RR intervals. Atrial tachycardia (AT) developed when the neonate was 23 days old. The key point that differentiates high-degree AV block from PACs with block is the PP interval. The PP interval is fixed in high-degree AV block and unfixed in PACs with block. Careful differential diagnosis is required in neonates with bradycardia because it may lead to very different management. Our case presents a good illustration of why these arrhythmias need to be differentiated. Furthermore, our case may be the first of neonatal lupus with AT.

## Introduction

Neonatal lupus erythematosus (NLE) is a syndrome characterized by clinical symptoms observed in neonates born to mothers with antibodies to soluble antigens of the cell nucleus (1). The primary factors contributing to the pathogenesis of this disease are anti-SSA/Ro and anti-SSB/La antibodies. These antibodies, after crossing the placenta, can trigger a cascade of inflammatory reactions (1). Approximately 50% of newborns with neonatal lupus are born to mothers with systemic lupus erythematous or Sjögren's syndrome (2). Many mothers are asymptomatic and anti-SSA or anti-SSB antibodies are only identified in them after skin rash or bradycardia occur in their babies, which are compatible with the diagnosis of neonatal lupus (3). The presentations of NLE are multiform. Non-cardiological symptoms are more frequent than cardiological symptoms and typically disappear within the first few months of life as the mother's antibodies clear from the infant's serum. However, 2% of affected infants develop irreversible complications, including disturbances in the cardiac stimulatory and conduction system (4). The characteristic annular or macular rash, typically involving the face and trunk, can be present at birth but more often appears within the first 6–8 weeks of life and may last for 3–4 months. Cytopenia and hepatitis may also be noted. These non-cardiac manifestations are usually transient. However, the most serious complication is permanent abnormalities in the cardiac conduction system. Cardiac manifestations consist of heart block, cardiomyopathy, valvular dysfunction, and endocardial fibroelastosis. Cardiac conduction system abnormalities range from a prolonged PR interval to complete heart block, which can be detected *in utero* using a fetal echocardiogram, beginning at a gestational age of 16 weeks, and may lead to hydrops fetalis (5). With a high mortality rate of approximately 20%, severe conduction system disturbances, such as high-degree or complete atrioventricular (AV) block, should be treated immediately with cardiac pacing (6–8). Therefore, it is important to recognize severe conduction system abnormalities in a fetus or neonate with bradycardia.

## Case description

The 3-day-old female neonate was delivered from a previously healthy woman with a birth body weight of 3,000 g at a gestational age of 37 + 2 weeks. Fetal arrhythmias were detected at a gestational age of 37 weeks. As soon as the obstetrician noticed the arrhythmias, he consulted us to obtain the opinion of pediatric cardiologists. Fetal echocardiography showed a normal heart rate (110–180 beats per minute) with an irregular rhythm. There was no pericardial effusion or fetal hydrops. Therefore, observation was suggested. As the baby was full-term, the obstetrician arranged for induction after discussing it with the mother and her family. After the mother was hospitalized, our pediatric cardiologist visited her and asked about her medical history. She said she had experienced dry eyes, dry mouth, and dry skin on her hands for years. There was no skin rash or joint pain. Because Sjögren's syndrome could not be ruled out, the obstetrician checked her antibodies. For the same reason, the antibodies of the newborn were also checked after birth.

On day 1, an electrocardiogram (ECG) revealed frequent premature atrial contractions (PACs). Echocardiography showed no structural heart disease, cardiomyopathy, or pericardial effusion. Laboratory data, including electrolytes, cardiac enzymes, blood gas, and thyroid function, were normal, except that the mother and neonate were both positive for anti-SSA/Ro antibodies. Neonatal lupus can be diagnosed based on positive maternal and neonatal anti-SSA/Ro antibody.

**Figure 1:**
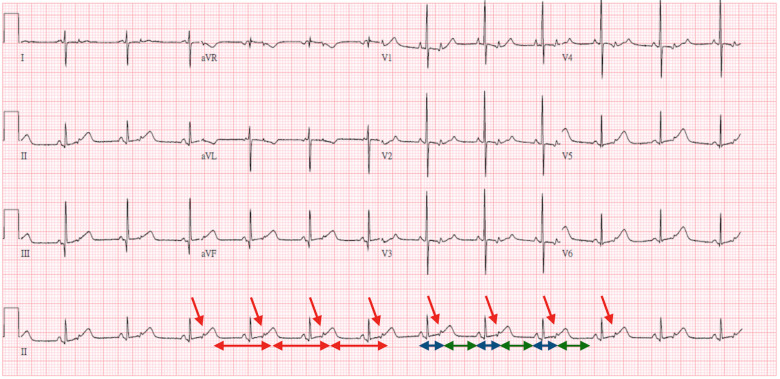
Three-day-old bradycardia with a heart rate of 70–75 beats per minute. ECG interpretation: The rhythm was regular; one P wave was followed by a narrow QRS and the other P wave was buried in the T wave (red arrow) without a following QRS. The RR interval and interval between every two conducted P waves (red line) were fixed. However, the interval between the conducted P wave and non-conducted P wave (blue line) was shorter than the interval between the non-conducted P wave and conducted P wave (green line), which implied that the P wave was contracted early and was not followed by a QRS. Therefore, bigeminy PACs with block was diagnosed.

Two days later, bradycardia (70–75 beats per minute) was noted. An ECG was performed and demonstrated bradycardia with a heart rate of 70–75 beats per minute (Figure 1). The rhythm was regular; one P wave was followed by a narrow QRS and the other P wave was buried in the T wave (red arrow) without a following QRS. Given the high degree of association between neonatal lupus and high-degree congenital heart block, this ECG was initially thought to indicate 2 to 1 AV block. In this ECG, the RR interval and interval between every two conducted P waves (red line) were fixed. However, the interval between the conducted P wave and non-conducted P wave (blue line) was shorter than the interval between the non-conducted P wave and conducted P wave (green line), which implied that the P wave was contracted early and without a following QRS. Therefore, bigeminy PACs with block was diagnosed.

**Figure 2:**
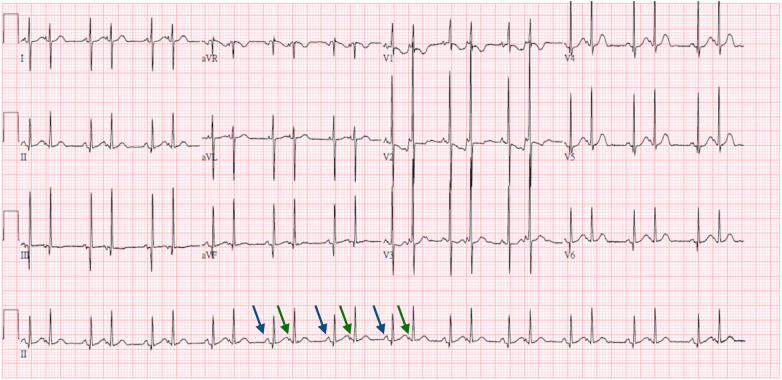
Seven-day-old bigeminy PACs with a heart rate of 150–170 beats per minute. ECG interpretation: The P wave of the sinus rhythm (blue arrow) was taller and broader than the P wave of the PACs (green arrow), which indicated the atrial contraction form atrial myocardium lower than SA node.

**Figure 3:**
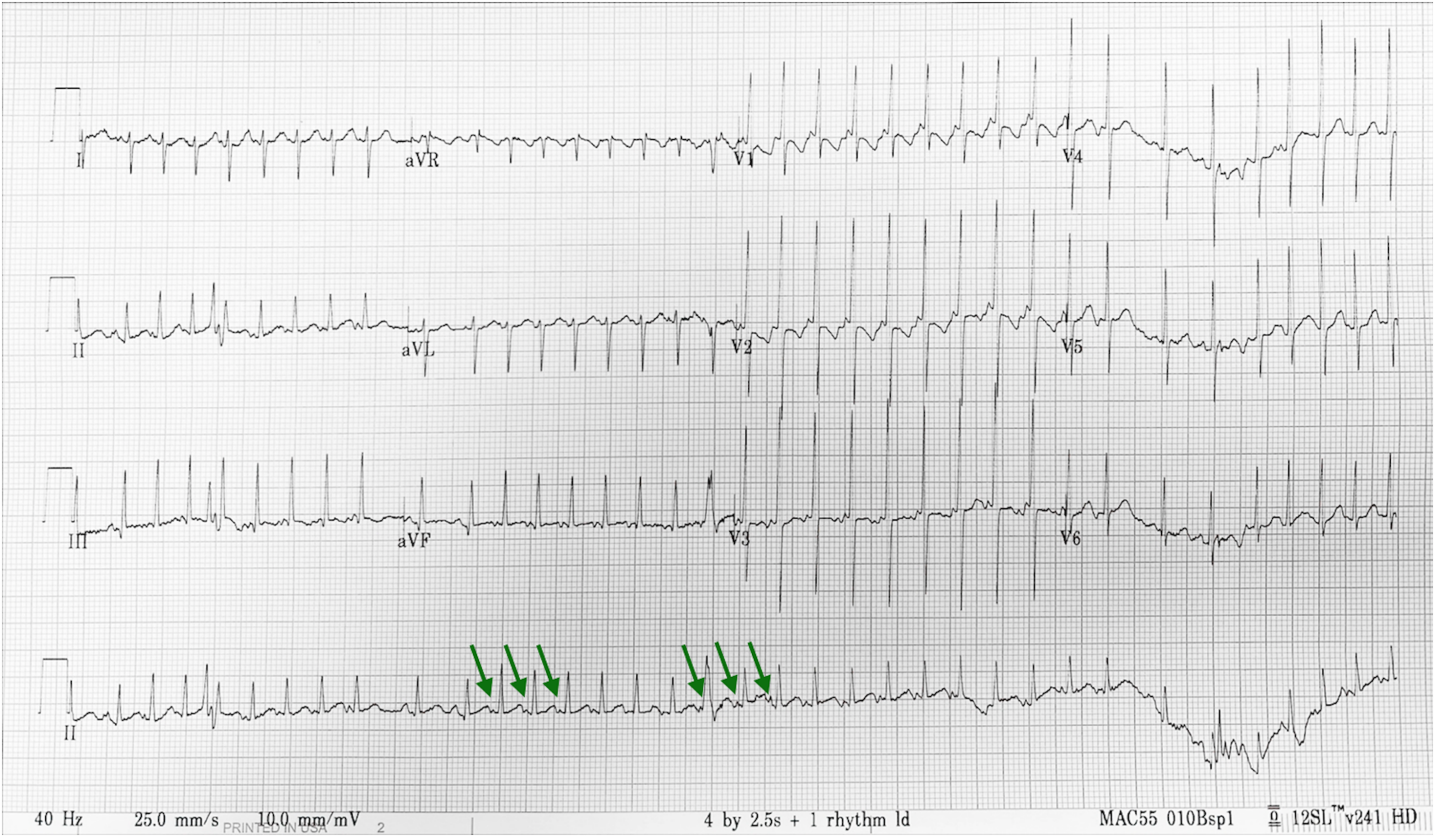
Twenty-three-day-old long RP and narrow QRS tachycardia with a heart rate of 200–250 beats per minute. ECG interpretation: The shorter and narrower P wave (green arrow) conducted form other atrial myocardium rather than SA node was still noted. Thus, atrial tachycardia was diagnosed.

At 7 days, bigeminy PACs with a heart rate of 150–170 beats per minute was noted (Figure 2), which has correlated with the diagnosis of bigeminy PACs with block previously. In this ECG, the P wave of sinus rhythm (blue arrow) was taller and broader than the P wave of PACs (green arrow), which indicated the atrial contraction form atrial myocardium lower than sinoatrial node (SA node). In addition, a long RP and narrow QRS tachycardia (200–250 beats per minute) were found (Figure 3) at 23 days old. In this ECG, the shorter and narrower P wave (green arrow) conducted form other atrial myocardium rather than SA node was still noted. Thus, atrial tachycardia (AT) was diagnosed and it resolved after infusion with amiodarone. After tachycardia terminated, amiodarone was discontinued gradually. Propranolol was added. The patient was then discharged and followed up through the outpatient department. At 6 months, the patient no longer had sustained tachycardia except for a few PACs. Propranolol will be tapered if the patient’s clinical condition remains stable.

## Discussion

The precise molecular mechanism through which anti-SSA/SSB antibodies affect the fetal skin and heart is still under investigation. The presence of anti-SSA/SSB antibodies is necessary but insufficient to cause heart block. The development of neonatal lupus involves the interaction between the transplacental passage of maternal antibodies and additional factors, such as fetal genetics [specific human leukocyte antigen (HLA) alleles] and environmental influences, which explains the rarity of these complications (9). Immunohistochemistry data confirm that the permanent electrogenic disturbances are ultimately caused by fibrosis and calcification, although the pathway to fibrosis may vary (9). Two main theories explain the molecular mechanism of autoimmune-induced heart block: the “apoptosis hypothesis” and the “calcium channel hypothesis” (9, 10). The apoptosis hypothesis theory suggests that the antibodies bind to surface antigens of apoptotic cells during physiological remodeling (9). Normally, intracellular antigens move to the surface of fetal cardiomyocytes during apoptosis. Immune complexes hinder the clearance of these apoptotic cells, leading to macrophage infiltration and cytokine release (TNF-α and TGF-β), which stimulates the differentiation of fibroblasts into myofibroblasts. These processes result in scar formation, contribute to inflammation and fibrosis, and then eventually lead to irreversible heart block (10). The calcium channel hypothesis theory states that the antibodies inhibit L-type and T-type calcium channels, which are essential for impulse propagation in the SA and AV nodes (10, 11). These processes lead to a decrease in calcium currents and the internalization of calcium channels, resulting in disruption to intracellular calcium handling, which then contributes to apoptosis, inflammation, and fibrosis (10). Neonatal lupus remains poorly understood with no clear care standards for pregnant women or postnatal care for mothers and infants. Existing studies, based on small populations from several countries, provide limited information on different populations (4).

Administering hydroxychloroquine to women with systemic lupus erythematosus (SLE) can reduce the risk of fetal heart block and anti-SSA/SSB-related cardiac complications in neonatal lupus (9). If the mother tests positive for anti-SSA/SSB antibodies, serial echocardiograms should be performed to detect early fetal abnormalities, such as PACs or pericardial effusion, which may precede a total AV block (4). There is still debate about the specific treatment for each degree of heart block, particularly regarding the prophylactic medication for first-degree block (9). Although no current guidelines exist, approximately 50% of specialists recommend starting steroids after a diagnosis of first-degree block (9). Depending on fetal echocardiography, dexamethasone may be continued up to 26 weeks of gestation, when the critical period reaches the end (9). A 2018 systematic review and meta-analysis of five observational studies concluded that the use of fluorinated glucocorticoids should not be discouraged for second-degree immune-mediated congenital heart block until more robust evidence is available (9, 11). Third-degree fetal block is irreversible. It implies fibrosis and calcification of the AV node (9). At this stage, the goal is to prevent fetal hydrops and intrauterine death, as many studies have proved that fluorinated glucocorticoids are ineffective (9, 12). Fluorinated steroids should not be used prophylactically in the absence of symptoms during the fetal period (4). Intravenous immunoglobulin is not used prophylactically but can increase the chances of restoring sinus rhythm if administered early enough after the detection of AV block (4). In the postnatal period, asymptomatic infants born to mothers with anti-SSA/SSB antibodies should be screened using ECGs (9). Intense monitoring is required if a first- or second-degree block is identified after birth, due to the risk of progression to third-degree block (9). Infants with complete heart block and heart rates under 55 beats per minute require the implantation of a cardiac pacemaker (9, 13, 14).

Cardiac conduction abnormalities observed in newborns with neonatal lupus include first-degree AV block, second-degree AV block, complete AV block, atrial and ventricular ectopic beats, atrial flutter, ventricular and junctional tachycardia, long QT syndrome, and sinus node dysfunction (15). Of these, high-degree AV block is the most notable because of its severity and high association with the anti-SSA/Ro antibody. Congenital cardiac blocks are diagnosable *in utero* or in the early neonatal period and 80%–90% are neonatal lupus related (16). Incomplete heart blocks as the initial cardiac presentation in neonatal lupus can progress to complete heart block at any age despite the absence of circulating antibodies (17).

In this case, the differential diagnosis between high-degree AV block and PACs with block is important because it may lead to very different management (Table 1) (16–18). High-degree AV block may progress to complete AV block and a pacemaker may be inserted. However, no emergent treatment is needed and only close observation is required for PACs with block. AV block can be classified into three types. First-degree AV block is just a conduction delay between the atrium and the ventricle. The P wave always precedes the QRS complex but the PR interval is prolonged without any dropped beat. There is an intermittent conduction block in second-degree AV block and it is further divided into two subgroups: Mobitz type I, in which the PR interval is prolonged gradually and followed by a non-conducted P wave, and Mobitz type II, in which the PR interval is fixed and not prolonged and suddenly followed by a non-conducted P wave. Mobitz type I and II can cause a distinct form fixed ratio AV block (2 to 1, 3 to 1, or 4 to 1). It is difficult to recognize and refer to high-degree AV block. Third-degree AV block is a complete conduction block; the atrium and ventricle contract independently, resulting in separate fixed PP and RR intervals. A PAC is a premature beat arising from an atrial myocardium other than the SA node. Consequently, the morphology of the P wave will be different from the morphology of a normal sinus P wave and the PP interval will be shorter than the last PP interval. If a PAC arrives early in the cardiac cycle, it may be conducted aberrantly (PAC with aberrancy) or not be conducted (PAC with block). Therefore, the key point that differentiates high-degree AV block from a PAC with block is the PP interval. The PP interval is fixed in high-degree AV block and not fixed in a PAC with block. Our case provides a good illustration of how to differentiate these arrhythmias.

**Table 1:** Comparison of high-degree AV block and PAC with block (16–18).

	High-degree AV block	PAC with block
Incidence	Occurs in 1%–3% of neonates whose mothers had positive autoimmune antibody (16)	Occurs in 51% of normal neonates (17)
Etiology	If without congenital heart disease, 90% born to mothers with autoimmune disorders (17)	May be related to electrolyte abnormalities, hypoglycemia, hypoxia, and hyperthyroidism (17)
ECG characteristics	PP and RR intervals are fixed separately	PP interval is not fixed due to earlier atrial conduction
Associated abnormalities	15%–20% associated with myocardial dysfunction (16)	Rarely associated with other abnormalities (17)
Mortality	8%–16% without treatment (18)	Generally benign (17)
Treatment	May implant pacemaker if there is ventricular dysfunction, prolonged QT, or a ventricular rate <55 bpm (16)	Maintain observation (17)

On the other hand, our case may reveal the association between AT and neonatal lupus. AT is a supraventricular tachycardia with an electrical impulse from somewhere in the atria other than the SA node. It is broadly categorized as focal (originating from a small circumscribed area) or macroreentrant (an uninterrupted activation wavefront rotating around a relatively large central obstacle). Acute therapy for focal AT consists of an intravenous beta-blocker and a calcium channel blocker (diltiazem or verapamil). Vagal maneuvers and adenosine may be used to terminate AT but the response is variable. For refractory AT, intravenous ibutilide, amiodarone, or class IC drugs (flecainide or propafenone) can be considered. Electrical cardioversion is reserved for hemodynamic unstable patients. Among the diversity of cardiac manifestations of neonatal lupus, AV block, atrial and ventricular ectopic beats, atrial flutter, ventricular and junctional tachycardia, long QT syndrome, and sinus node dysfunction have been reported except for AT. Our case may be the first of neonatal lupus with AT. However, this connection requires further observation and clinical evidence for confirmation.

## Data Availability

The raw data supporting the conclusions of this article will be made available by the authors, without undue reservation.
